# A prevalent *MOCS2* variant in the Roma population is associated with a novel mild form of molybdenum cofactor deficiency

**DOI:** 10.1007/s00431-025-06335-x

**Published:** 2025-07-25

**Authors:** Sung Kweon Cho, Guenter Schwarz, Velibor Tasic, Michaela Křížková, Jakub Krijt, Juliane Roeper, Tomáš Honzík, Ivan Šebesta, Viktor Kožich, Jana Šaligová, Katerina Pavelcova, Jana Masinova, Cheryl A. Winkler, Blanka Stiburkova

**Affiliations:** 1 Department of Pharmacology, Ajou University School of Medicine, 164, World Cup-Ro, Yeongtong-Gu, Suwon, South Korea; 2 Molecular Genetic Epidemiology Section, Basic Research Laboratory, National Cancer Institute, Frederick, MD USA; 3 Institute of Biochemistry, Department of Chemistry and Center for Molecular Medicine, University of Cologne, Cologne, Germany; 4 Faculty of Medicine, University Ss. Cyril and Methodius, 1000 Skopje, North Macedonia; 5 Department of Pediatrics and Inherited Metabolic Disorders, First Faculty of Medicine, Charles University and General University Hospital, Prague, Czech Republic; 6 Children’s Faculty Hospital Kosice, Košice, Slovakia; 7 Institute of Rheumatology, Na Slupi 4, 128 50, Prague, Czech Republic; 8 Cancer Innovation Laboratory, Center for Cancer Research, National Cancer Institute, Frederick, MD USA; 9 Department of Rheumatology, First Faculty of Medicine, Charles University, Prague, Czech Republic

**Keywords:** MOCS, MoCD, Xanthinuria, Genetic screening test, Sulfite, Uric acid

## Abstract

Xanthinurias are rare inherited disorders of purine metabolism. Xanthinuria type III is caused by molybdenum cofactor deficiency (MoCD) due to pathogenic variants in *MOCS1*, *MOCS2*, *MOCS3,* or *GEPH* genes*.* Here, we described five Roma patients from four unrelated families with hypouricemia, accumulation of xanthine/hypoxanthine, deficiency of xanthine oxidase activity, variable age of diagnosis, and only asymptomatic or mild clinical course. Whole exome sequencing was performed on all probands, aged 3 to 43 years, due to lack of genetic confirmation for xanthinuria types I and II. The causality of the putative pathogenic variant was confirmed by analysis of sulfite and related metabolites and in vitro functional characterization of metal-binding pterin (MPT) synthesis and protein complex formation. Considering the rarity of the condition and recessive inheritance, 34 candidate variants were identified after filtering out allele frequency threshold in non-Finnish Europeans. An ultra-rare *MOCS2* variant rs776441627 in two overlapping reading frames (c.244A > T (NM_176806.4; p.Ile82Phe) = c.57A > T (NM_004531.5; p.Leu19Phe)) segregated with the disease in all five patients (four homozygotes, one compound heterozygote). The variant has an allele frequency of 3.6% in a Roma population control group. Functional characterization revealed the significantly decreased MPT synthesis activity and confirmed the causality of rs776441627 in MoCD.

*Conclusion*: The rs776441627 is a functional variant for MoCD with a mild to asymptomatic clinical phenotype and fully penetrant biochemical phenotype. Hypouricemia should be considered in the differential diagnostic algorithm of pediatric and adult patients with neurological symptoms, and *MOCS2* should be considered in gene panels for xanthinuria screening.

**Table:** 

**What is Known:** • *Xanthinuria type III is caused by molybdenum cofactor deficiency (MoCD) due to pathogenic variants in MOCS1, MOCS2, MOCS3, or GEPH genes.* • *The majority of patients with xanthinuria III present with classical early-onset MoCD due to autosomal recessive variants in the MOCS1 gene, manifesting severe progressive neurological complications during the first postnatal days.* • *To date, approximately 40 patients with MoCD due to pathogenic MOCS2 variants have been reported; most were diagnosed during the neonatal period with intractable seizures and feeding disorders.*	
**What is New:** • *A novel ultra-rare variant, rs776441627, located in two overlapping reading frames of the MOCS2, was identified in five Roma patients presenting a mild to asymptomatic clinical MoCD phenotype and a fully penetrant biochemical phenotype.* • *Functional studies of p.Ile82Phe (small MOCS2A subunit) and p.Leu19Phe (large MOCS2B subunit) demonstrate a strong reduction in molydopterin synthase complex formation and activity, consistent with the changes in biomarkers of MoCD observed in affected individuals.* • *The rs776441627 variant shows significantly elevated frequency among the Roma population, highlighting the importance of considering ethnic background in the differential diagnosis of MoCD.* • *Hypouricemia may provide an initial, generally available biochemical key marker indicator of molybdenum cofactor deficiency.*

**Supplementary Information:**

The online version contains supplementary material available at 10.1007/s00431-025-06335-x.

## Introduction

Xanthinurias are rare autosomal recessive disorders of purine metabolism characterized by severe hypouricemia and accumulation of xanthine and hypoxanthine in body fluids and tissues. Type I (OMIM 278300) is caused by xanthine dehydrogenase/oxidase deficiency (XDH/XO, EC 1.17.1.4/1.17.3.2) [1]; type II (OMIM 603592) results from a combined deficiency of XDH/XO and aldehyde oxidase (AOX, EC:1.2.3.1) caused by dysfunctional variants in the molybdenum cofactor sulfurase (MOCOS, EC 2.8), which is required for XDH/XO and AOX activity [2]. Types I and II xanthinuria are the classical forms of xanthinuria presenting with urolithiasis/nephrolithiasis and deposits of xanthine in various organs in about half of patients while the other half is asymptomatic [3]. The clinically distinct, third type of xanthinuria (OMIM 252150) is caused by defects in molybdenum cofactor (MoCo) synthesis leading to MoCo deficiency (MoCD). MoCo is the active site cofactor of both XDH/XO and AOX and is required for the activity of sulfite oxidase [4]. Sulfite oxidase deficiency leads to an accumulation of neurotoxic compounds such as sulfite, S-sulfocysteine (SSC(and S-sulfohomocysteine (SSH) [5, 6], and a decrease of total cysteine and homocysteine. MoCD exhibits a wide spectrum of presentations from severe early-onset disease to a milder late-onset form. The early-onset form of MoCD (caused by MOCS1 deficiency, also called MoCD-A) typically presents as neonatal encephalopathy with intractable seizures, brain atrophy, lens dislocation, and a high mortality in infancy. The less frequent late-onset forms of MoCD due to biallelic pathogenic variants in MOCS2 (also called MoCD-B) manifest in infancy to early childhood; patients have a history of normal cognitive development or mild developmental delay with hypotonia before first coming to medical attention, which frequently occurs as a result of acute neurologic deterioration [7]. Patients with both forms of MoCD may also present with lens dislocation.

**Fig. 1:**
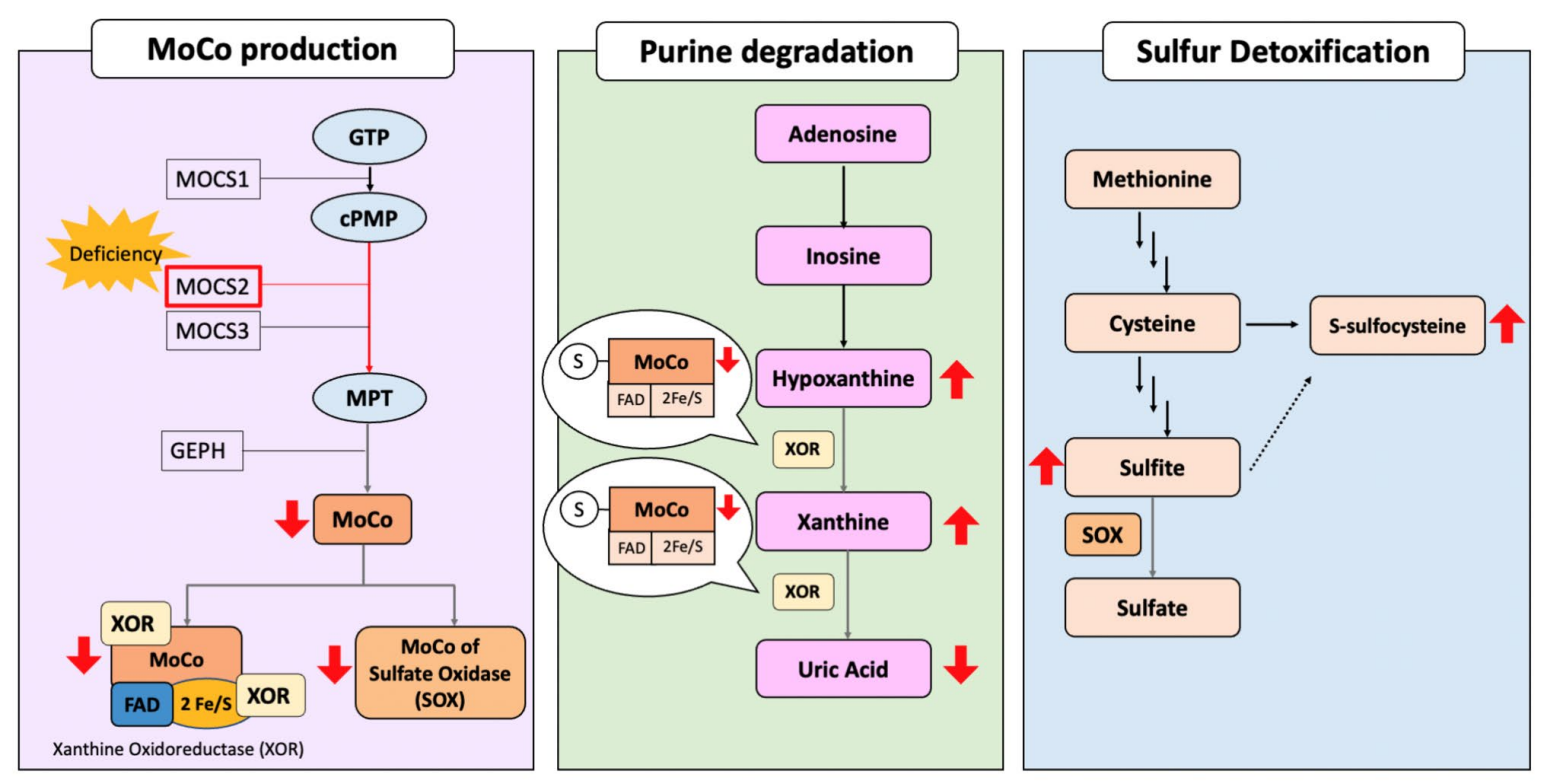
Mechanism of MoCo biosynthesis and purine degradation/cysteine catabolism

MoCo biosynthesis is a highly conserved biochemical pathway involving four genes: *MOCS1* (OMIM 603707), *MOCS2* (OMIM 603708), *MOCS3* (OMIM 609277), and *GEPH* (OMIM 603930) (Fig. 1) [8]. Patients diagnosed with MoCD are classified into three groups according to the affected step of biosynthesis: Type A, caused by a defect in *MOCS1*; Type B, caused by a defect in *MOCS2 and MOCS3*; and Type C, caused by a defect in the *GEPH* gene, which disrupts the formation of inhibitory synapses [9]. *MOCS1* ensures the synthesis of cyclic pyranopterin monophosphate from guanosine triphosphate [10], *MOCS2* catalyzes the conversion of cyclic pyranopterin monophosphate to molybdopterin [11], while gephyrin |(encoded by *GEPH*) facilitates the insertion of the molybdenum atom to form MoCo [12].

A total of 40 different variants were previously identified in 100 patients with MoCD [13–15]. According to published case reports and review articles, the majority of the patients present with classical early onset MoCD due to autosomal recessive variants in the *MOCS1* gene. *MOCS1* variants presented as severe and progressive neurological disorders; only 14 cases with the atypical presentation of late onset, less severe clinical symptoms, and basal ganglia dysfunction have been identified [16–18]. Approximately 1/3 of MoCD patients were diagnosed with MoCD type B due to variants in the *MOCS2* gene. Although MoCD type B patients generally present with severe phenotypes, the relationship between disease severity and molybdopterin synthase activity, and the phenotypic expression of different missense variants remains largely unexplored.

Here, we report five patients, aged 3 to 43 years, exhibiting severe hypouricemia, xanthinuria, and intellectual disability without seizures or severe phenotypes. Since Sanger sequencing of the *XDH/XO*, *AOX*, and *MOCOS* genes did not reveal any pathogenic variants, we subsequently performed whole exome sequencing (WES) which revealed a novel causal variant; further investigation using in silico prediction and functional analysis confirmed causality and expanded the knowledge on the phenotypic spectrum of MoCD.

## Materials and methods

### Subjects

In this study, we carried out a detailed biochemical, enzymatic, and genetic analysis in five Roma patients from four apparently unrelated families residing in Czechia, Slovakia, and Macedonia: proband 1 (family 1), proband 2 (family 2), probands 3 and 4 (family 3), and proband 5 (family 4). Inclusion criteria included biochemically confirmed xanthinuria. Patients or their guardians signed an informed consent for biological sample collection, storage, and genetic testing.

The allele frequency of the *MOCS2* variant identified in the patients was subsequently investigated in a population control cohort of 167 unrelated subjects of Roma ethnicity from Czechia and Slovakia enrolled in the hypouricemia cohort; Roma ethnicity was self-declared. A previously reported cohort of genomic DNA of 109 unrelated subjects of Roma ethnicity (chosen irrespective of their state of health) from Macedonia was used as a second control group [19]. All tests were performed following standards set by institutional ethics committees; the study was approved by the Ethics Committee of the Institute of Rheumatology on 24 July 2018 (project no. 7131/2018). All procedures were performed following the Declaration of Helsinki. The samples of biological materials were collected in the Bank of Biological materials of the Institute of Rheumatology.

### Biochemical and metabolite analysis

Creatinine (Cr) in plasma and urine was measured using the Jaffé reaction adapted for an auto-analyzer (Hitachi Automatic Analyzer 902, Roche, Basel). Uric acid in serum and urine was measured using a specific enzymatic method on the same auto-analyzer. High-performance liquid chromatography (HPLC) determination of hypoxanthine and xanthine in urine was performed on Waters Alliance 2695 with a Photodiode Array Detector 2998 (Waters, Massachusetts, USA) as described previously [20]. Sulfur-containing metabolites in lithium heparin plasma and freshly voided urine were determined as described previously [6]. Briefly, plasma and urinary total cysteine and homocysteine were determined by reversed-phase HPLC with fluorescent detection after reduction of disulfides and protein-bound species with tris(2-carboxyethyl)phosphine [21]. Inorganic anions sulfite and thiosulfate (SO_3_^2−^, S_2_O_3_^2−^) were determined by HPLC after derivatization with monobromobimane; S-sulfocysteine and S-sulfohomocysteine were determined by LC–MS/MS [22, 23]. Xanthine oxidase activity in plasma was determined using a modified method as published previously [24].

### Genetic diagnostic approach

Genomic DNA was extracted from ethylenediaminetetraacetic acid (EDTA) whole blood using a QIAmp DNA Mini Kit (Qiagen, Crawley, UK). All exons of *XDH/XO*, *AOX1*, and *MOCOS* genes were amplified by PCR and purified using a PCR DNA Fragments Extraction Kit (Geneaid, Taiwan). DNA sequencing was performed with a DNA sequencer (Applied Biosystems 3130 Genetic Analyzer; Applied Biosystems, USA). The sequencing analysis covered 36 exons and intron–exon boundaries for *XDH/XO* [24, 25], 35 exons for *AOX1*, and 15 exons for *MOCOS* [26]. The following resources were used in the genomic and protein sequences analyses of genes: *XDH/XO* (Ensembl ENSG00000158125, UniProt P47989), *AOX1* (Ensembl ENSG00000138356, UniProt Q06278), *MOCOS* (Ensembl ENSG00000075643, UniProt Q96EN8). Clinical WES approach was applied to undiagnosed patients (proband 1, proband 2, proband 3) and parents of probands 2 and 3. After identifying *MOCS2* as a putative causal variant, the exons and splicing site of *MOCS2* in probands 2, 3, 4, and 5 were confirmed using direct DNA sequencing (Metabolic Laboratory, VUMC, Amsterdam, Netherlands).

### Whole exome sequencing and variant filtering analysis

Genomic DNA isolated from blood lymphocytes was subjected to exome capture, using Agilent SureSelect target enrichment probes (Santa Clara, CA) and human exome capture arrays (Life Technologies, Carlsbad, CA) followed by next generation sequencing on the Illumina HISEQ 2500 sequencing system (Illumina, San Diego, CA). Sequencing was performed with 2 × 100 bp read length. Sequence reads were mapped to the human reference genome assembly (GRCh37/hg19). All variants were called and annotated using CLC Genomic Workbench (version 9.0.1) software (QIAGEN, San Diego, CA). The depth coverage was 85-fold. The overall variant-identifying process followed the standard guidelines of investigating variants for Mendelian disorders from WES data by the American College of Medical Genetics and Genomics [27, 28]. We performed the analysis assuming autosomal recessive inheritance mode as observed previously in xanthinuria [1, 2, 29], and the analysis pipeline was used as previously described [30].

First, based on the prevalence of xanthinuria, the Hardy–Weinberg equation was used to calculate the allele frequency threshold to 0.0005; we excluded variants with MAF > 0.05% in dbSNP database (version 150), 1000 Genome Projects phase 3 data (2504 individuals), Exome Aggregation Consortium (ExAC; http://exac.broadinstitute.org), and Genome Aggregation Database (gnomAD, http://gnomad.broadinstitute.org/) [31]. Second, variants present in the homozygous or heterozygous state in Non-Finnish Europeans were excluded. Third, non-synonymous variants, insertion/deletion (indel), or splice site variants were selected. In the final analysis, we excluded single heterozygous variants such that only homozygous variants and putative compound heterozygous variants remained.

### In silico* analysis*

Remaining variants from the filtering algorithm were ranked based on their impact score and extent of amino acid conservation across vertebrate orthologs using the UCSC Genome Browser (https://genome.ucsc.edu/). AVIA (Annotation, Visualization and Impact Analysis of genomic variants and genes) was used for annotation and in silico prediction [32]. This approach includes the latest versions of PolyPhen-2, SIFT, Variant Taster algorithms, Functional Analysis through Hidden Markov Models, and CADD [33–37]. Variant classification followed the guidelines of the American College of Medical Genetics and Genomics [38]. The candidate variant was confirmed by Sanger sequencing as previously described [30].

### Kinship coefficient calculation and age of MOCS2 variant

The kinship coefficient of two individuals is the measurement of relatedness. It is defined as the probability of a pair of randomly sampled homologous alleles being identical by descent to a gene drawn at random from the same locus from an unrelated subject. We used the PLINK program (v. 1.9) for the calculation and chose the KING-robust kinship estimator [39]. We compared kinship coefficients of a group of participating individuals and their family members.

### Functional characterization of MOCS2A p.Ile82Phe and MOCS2B p.Leu19Phe variants

MOCS2A and MOCS2B wild-type (WT) proteins and MOCS2A p.Ile82Phe and MOCS2B Leu19Phe variants were recombinantly expressed in *Escherichia coli* and purified to homogeneity as described previously [40]. The small subunits of MPT synthase, MOCS2A WT and MOCS2 p.Ile82Phe, were expressed and purified as intein-fusion proteins with a chitin-domain for subsequent affinity purification. These were eluted with ammonium sulfide, resulting in the release of activated MOCS2A proteins with a thiocarboxylated C-terminal tail [11]. The MOCS2B WT and the MOCS2B p.Leu19Phe variant were cloned into pET15b, expressed in *Escherichia coli* BL21. Purification was by ammonium sulfate precipitation and subsequent size exclusion chromatography using Superdex 200 column equilibrated in 100 mM Tris buffer, pH 7.5, 200 mM NaCl. Changes in the three-dimensional structure of the mutant protein were analyzed by circular dichroism spectroscopy using 10–30 µM protein in 10 mM potassium phosphate buffer, pH 7.5 at 20 °C.

Complex formation of MPT synthase was analyzed by isothermal titration calorimetry with a VP-ITC device (MicroCal, Freiburg, Germany) with 20–50 µM MOCS2B protein placed in the sample cell (1.5 mL) and 200–500 µM MOCS2A proteins in the syringe for titrating into the sample cell to determine binding parameters. Before performing the experiment, proteins were dialyzed against ITC buffer (10 mM Tris–HCl, 250 mM NaCl, pH 8.0). The binding enthalpy was directly measured, while the association constants (*K*_a_) and stoichiometries (*N*) were obtained by data analysis using ORIGIN software.

In vitro MPT synthesis rates were quantified as a function of the concentration of small MPT synthase subunit MOCS2A as described before [41].

## Results

### Biochemical and metabolite results

**Fig. 2:**
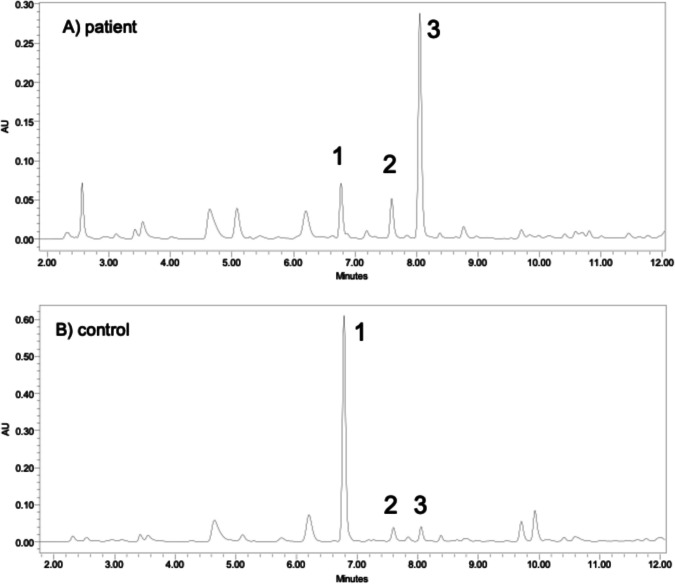
HPLC result in urine samples (260 nm). **A** Patient with molybdenum cofactor deficiency (MoCD) (proband 1); **B** healthy control; 1. Uric acid, 2. Hypoxanthine, 3. Xanthine

Biochemical results in the blood and urine of all patients are summarized in Table 1. Representative HPLC–UV chromatograms of urine samples in a control subject and proband 1 are shown in Fig. 2.

**Table 1:** Biochemical results of probands with xanthinuria type III

	Analyte [µmol/L]	Proband 1	Proband 2	Proband 3	Proband 4	Proband 5	Reference range
**Blood**							
Sulfur containing compounds^1^	Total homocysteine	**1.1**	**2.6**	**3.3**	**2.3**	**1.8**	4.9–14.9
	Total cysteine	**78**	N/A	N/A	**92**	**95.2**	155–334
	S-sulfocysteine	**10.4**	N/A	N/A	**4.4**	**7.2**	0.5–1.8
	Sulfite	**5.2**	N/A	N/A	**7.5**	**7.4**	0.2–0.6
	Thiosulfate	**3.1**	N/A	N/A	**7.7**	**9.3**	0.1–1.1
Purines^2^	Uric acid	**70**	50	49	**33**	**32**	120–360
Enzymology^3^	XDH/XO activity^4^	**0**	N/A	N/A	**0**	N/A	17–128
	Analyte [mmol/mol Cr]	Proband 1	Proband 2	Proband 3	Proband 4	Proband 5	Control range
**Urine**							
Sulfur containing compounds	Total homocysteine	0.6	N/A	N/A	2.1	1.2	0.3–2.5
	Total cysteine	26.7	N/A	N/A	69.0	64.1	8.9–88.4
	S-sulfocysteine	**52**	N/A	N/A	**67**	**47**	0.4–0.9
	Sulfite	**6.7**	N/A	N/A	**102**	**386**	0.02–0.2
	Thiosulfate	**19.7**	**179.5**	**110.9**	**189**	**166**	0.8–3.1
Purines	FE-UA*	**5.8**	**2.6**	**2.0**	**5.0**	N/A	6.0–8.6
	Xantine	**215**	**221**	**367**	**327**	**344**	< 30
	Hypoxanthine	**60**	**88**	**168**	**216**	**43**	< 25

Abbreviations: *FE-UA* fractional excretion of uric acid (%), *N/A* not available

^1^LiHep Plasma

^2^Serum

^3^EDTA Plasma

^4^[nmol/hour/L plasma]

In the plasma samples, the concentrations of total homocysteine, total cysteine, urate, and XDH/XO activity were decreased. In contrast, the levels of sulfite-related metabolites—specifically SSC, sulfite, and thiosulfate—were markedly elevated.

In urine samples, no significant changes were observed in total homocysteine and total cysteine levels. However, sulfite-related compounds (SSC, sulfite, and thiosulfate) and purine degradation products (xanthine and hypoxanthine) were significantly elevated compared to the control.

Overall, probands exhibited a distinct biochemical profile characterized by markedly increased concentrations of sulfite-related compounds in blood and urine, along with an excessive accumulation of xanthine-class compounds in urine. Notably, no xanthine oxidation activity was detected. The clinical characteristics of all probands are summarized in Table 2.

**Table 2:** Clinical characteristics

Disease	Proband 1	Proband 2^1^	Proband 3^1^	Proband 4	Proband 5
Age at diagnosis	42 years	5	4	10	1
Sex	Female	Male	Female	Female	Female
History of consanguinity	No	No	No	No	No
Failure to thrive	No	No	No	Yes	No
short stature	Yes	No	Yes	Yes	No
Seizures	No	No	No	No	No
Onset age	N/A	2 years	4 years	5 years	N/A
Intellectual disability	Yes	Yes	No	Yes	Yes
Hyperkalemia	No	No	No	No	No
EEG	N/A	Normal	N/A	Normal	N/A
Head circumference	Normal	Normal	Normal	Microcephaly (− 2.81 SD)	Normal
Facial dysmorphism	No	No	No	No	No
Axial dystonia	No	No	No	No	No
Spastic quadriparesis	No	No	No	No	No
Extrapyramidal	No	No	No	No	No
Lens dislocation	No	No	No	No	No
Renal stone	Yes	No	No	No	No
Cardiomyopathy	No	No	No	No	No
Brain imaging calcification	No	N/A	N/A	No	N/A
Subcortical cysts	No	N/A	N/A	No	N/A
Abnormal basal ganglia	No	N/A	N/A	No	N/A
Cerebral atrophy	No	N/A	N/A	No	N/A
Thinned corpus callosum	No	N/A	N/A	No	N/A
White matter volume loss	No	N/A	N/A	No	N/A
Subcortical cysts	No	N/A	N/A	No	N/A
Abnormal basal ganglia	No	N/A	N/A	No	N/A
Cerebellar atrophy	No	N/A	N/A	No	N/A
Strabism, amblyopia, lens dislocation	No	No	No	Yes	No

Abbreviation: *N/A* not available

^1^Probands 2 and 3 are affected siblings

Proband 1 (family 1), proband 4 (family 3), and proband 5 (family 4) have persistent hypouricemia and elevated urinary excretion of hypoxanthine and xanthine (see Table 1 for details). The analysis of sulfur-containing compounds in lithium heparin plasma showed low total cysteine and homocysteine and markedly elevated SSC, sulfite, and thiosulfate. Xanthine oxidase activity in plasma was undetectable, and the absence of the metabolite oxypurinol in plasma after the allopurinol loading test confirmed the diagnosis of xanthinuria.

Proband 2 (family 2) had persistent hypouricemia with a lower excretion fraction of uric acid and elevated urinary excretion of hypoxanthine and xanthine (see Table 1 for details). Affected sibling (proband 3) had persistent hypouricemia, a lower excretion fraction of uric acid, and elevated urinary excretion of hypoxanthine and xanthine. Unfortunately, samples from family 2 were not available for laboratory testing for sulfur metabolites.

### Genetic diagnostic approach

WES was performed to characterize xanthinuria type, after failing to identify putative pathogenic variants in *XDH/XO*, *AOX1*, and *MOCOS* in proband 1. WES analysis identified 22 novel candidate missense variants (Supplementary Table S1). Among those, the nucleotide rs776441627 in the overlapping region of the *MOCS2* two open reading frames introduced a codon change in both the small MOCS2A subunit (p.Ile82Phe) and in the large MOCS2B subunit (p.Leu19Phe), respectively. Both subunits assemble into a hetero-tetrameric molybdopterin synthase complex consisting of two small (MOCS2A) and two large subunits (MOCS2B) [42]. The nucleotide exchanges at c.244A > T (*MOCS* 2 A) and c.57A > T (*MOCS*2B) both result in a conservative replacement of a hydrophobic amino acid side chain with another hydrophobic side chain in a highly conserved region of the A subunit and moderately conserved motif in the B subunit. These variants are extremely rare (0.00002475, gnomAD accessed Jan 3, 2025) and are predicted as likely pathogenic based on the occurrence of these gene products in highly conserved and moderately conserved regions of subunits A and B, respectively.

We further conducted WES in probands 2, 3, 4, and 5 and their parents. Homozygosity of *MOCS2A* c.244A > T segregated with xanthinuria in these families. In family 2, siblings 2 and 3 were homozygous for c.244A > T (NM_176806.4) = c.57A > T (NM_004531.5), whereas their unaffected parents and older sister were heterozygotes. Probands 4 and 5 were also homozygous for this *MOCS2* variant. Direct sequencing of *MOCS2* was performed in all probands for confirmation (Supplementary Fig. S1). We selected this variant as a putative causal variant based on its extreme rarity in super-populations, segregation with disease in multiple families, and the essential role of the gene in the biosynthesis of MoCo as per ACMG guidelines [38].

### Kinship coefficient and MAF in Roma population

Kinship coefficients for all affected individuals and their related family members are higher than 0.25, indicating a parent–child relationship or full sibling relationship (Table 3). Family 3 (father) and family 4 (mother) are related, and proband 3’s father and mother are closely related.

**Table 3:** Kinship coefficient

ID	Proband 4	Mother of proband 5	Mother of proband 4	Proband 5	Brother of proband 4	Father of proband 5	Father of proband 4	Proband 1	Father of probands 2 and 3	Mother of probands 2 and 3	Proband 2	Proband 3
Sister of probands 2 and 3	− 0.035	− 0.030	− 0.037	− 0.047	− 0.047	− 0.053	− 0.039	− 0.368	0.179	0.196	0.155	0.204
Proband 3	− 0.099	− 0.102	− 0.105	− 0.116	− 0.105	− 0.114	− 0.099	− 0.348	0.172	0.169	0.163	
Proband 2	− 0.092	− 0.097	− 0.106	− 0.101	− 0.101	− 0.108	− 0.094	− 0.326	0.169	0.174		
Mother of probands 2 and 3	− 0.045	− 0.048	− 0.053	− 0.055	− 0.055	− 0.062	− 0.051	− 0.315	− 0.055			
Father of probands 2 and 3	− 0.087	− 0.085	− 0.097	− 0.097	− 0.101	− 0.100	− 0.086	− 0.346				
Proband 1	− 0.372	− 0.349	− 0.389	− 0.344	− 0.380	− 0.358	− 0.353					
Father of proband 4	0.268	0.018	0.052	− 0.001	0.250	0.000						
Father of proband 5	− 0.004	0.001	− 0.012	0.229	− 0.010							
Brother of proband 4	0.230	0.011	0.256	− 0.007								
Proband 5	− 0.005	0.240	− 0.008									
Mother of proband 4	0.256	0.011										
Mother of proband 5	0.014											

Kinship coefficient > 0.25: parent–child or full sibling; > 0.125: half sibling; > 0.0625: first cousin

All of these patients with c.244A > T (NM_176806.4) = c.57A > T (NM_004531.5) variant self-identified as Roma. Next, we genotyped 167 controls from the Roma population in the Czech and Slovakia Republic and determined an allele frequency of 3.6% which was approximately 1500-fold enriched as compared to the frequency from gnomAD. On the other hand, in a Macedonian sub-cohort of 109 subjects of Roma ethnicity, the variant was not found.

### Functional characterization of c.244A > T (NM_176806.4) = c.57A > T (NM_004531.5) variant

To explore the functional impact of the respective mutations on MOCS2A and MOCS2B proteins, the variants were recombinantly expressed in *Escherichia coli* and purified to homogeneity. MOCS2A was expressed as an intein-fusion protein, cleaved by ammonium sulfide treatment on the column to obtain thiocarboxylated protein [11] and further purified by size exclusion chromatography (Supplementary Fig. S2A, C). MOCS2B was purified using ammonium sulfate precipitation and size exclusion chromatography (Supplementary Fig. S2B, C). CD-spectroscopy confirmed that neither of the variants showed any major alterations in their secondary structure composition, suggesting correct folding of the proteins (Supplementary Fig. S2D, E).

**Fig. 3:**
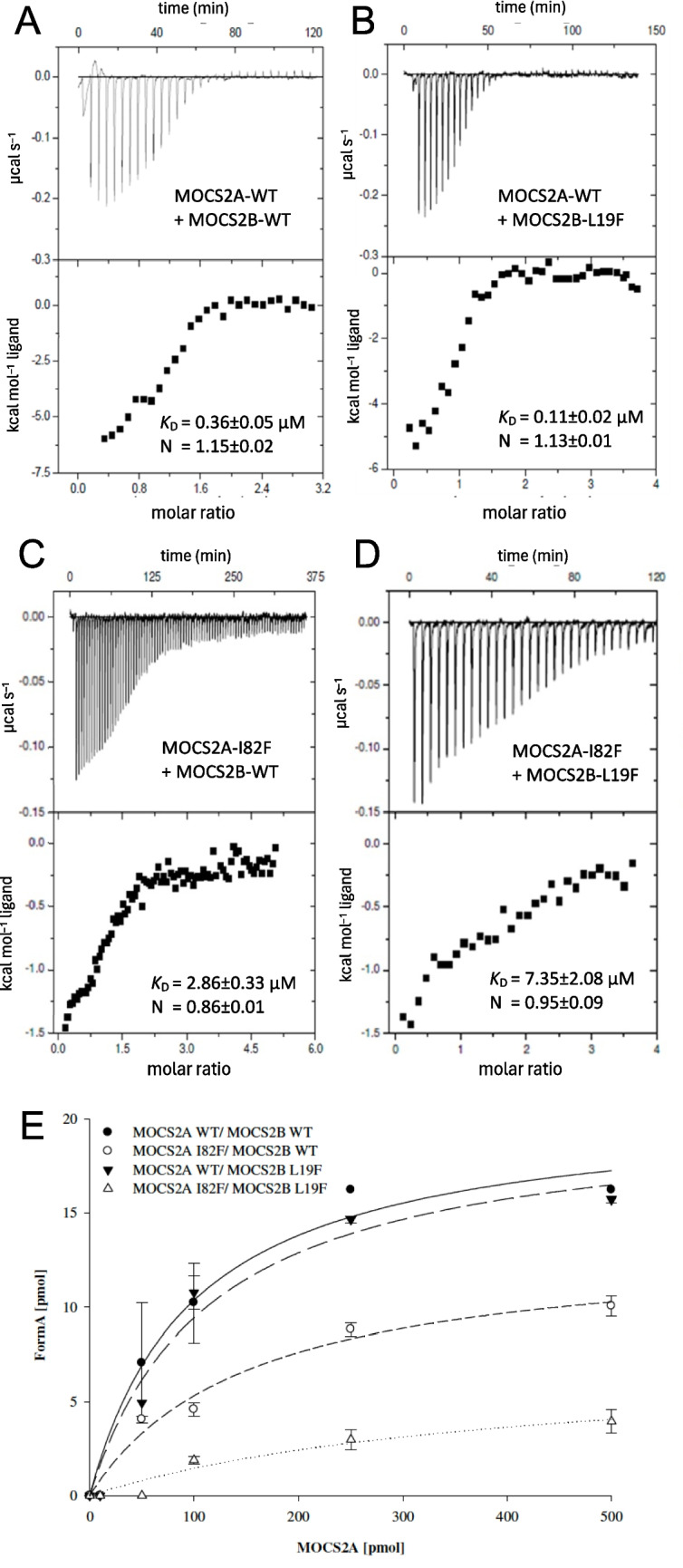
Functional characterization of MOCS2A p.Ile82Phe (I82F) and MOCS2B p.Leu19Phe (L19F) variants. **A**–**D** Isothermal titration calorimetry (ITC) of MOCS2A WT with MOCS2B WT (**A**) and MOCS2B L19F (**B**), MOCS2A I82F with MOCS2B WT (**C**) and MOCS2B L19F (**D**). In the upper graph, binding isotherms are shown; below are plots of the integrated isotherms versus the molar ratio of MOCS2A variant to substrate MOCS2B variant and fitted with ORIGIN. Calculated binding parameters for each condition are shown in each inset and are derived from *n* = 3 experiments. **E** In vitro MPT synthesis by MOCS2B variants as a function of MOCS2A variant with cPMP. Formed MPT has been determined by MPT Form A analysis

First, we determined the ability for both subunits to form a tetrameric molybdopterin synthase complex using isothermal titration calorimetry (ITC). WT MOCS2A and MOCS2B showed equimolar (*N* = 1.15 ± 0.2) interaction with a *K*_d_ of 359 ± 47 nM (Fig. 3A). Surprisingly, binding of WT MOCS2A to MOCS2B p.Leu19Phe variant showed an even higher affinity with a *K*_d_ of 114 ± 16 nM (Fig. 3B). In contrast, when using MOCS2A p.Ile82Phe variant, the strength of interaction dropped by one order of magnitude (*K*_d_ = 2.8 ± 0.3 µM, Fig. 3C) and was the lowest when both variants were combined (*K*_d_ = 7.4 ± 2.1 µM, Fig. 3D). Therefore, we conclude that both variants were able to form MPT synthase complexes with equimolar stoichiometry. However, the strength of interaction was approximately 20-fold reduced for the patient-derived mutant subunits MOCS2A p.82Phe and MOCS2B p.19Phe when compared to WT.

Next, we compared the in vitro MPT synthesis activity of both variants separately to the WT MOCS2A/B (Fig. 3E). We used 500 pmol purified cPMP [43] and incubated it with 200 pmol of the respective MOCS2B protein with 0–500 pmol of MOCS2A and determined the amount of synthesized MPT using the HPLC Form A method [44]. We found a WT-like activity for MOCSB p.Ile19Phe variant, while the activity was 50% reduced for WT MOCS2B with MOCS2A p.Ile82Phe and further reduced to approx. 20% of WT when both variants were combined. These data collectively demonstrate a strong reduction in MPT synthase complex formation and MPT synthesis activity, being consistent with the changes in biomarkers of MoCD observed in the patient.

## Discussion

MoCD are ultra-rare inherited disorders with approximately 200 cases reported worldwide [15]; the main pathogenic mechanism is due to the accumulation of toxic levels of sulfite resulting from deficiency of sulfite oxidase. Most published cases show classical presentation during the first postnatal days, including intractable seizures, feeding difficulties, severe encephalopathy, apnea, and axial hypotonia [13–15, 45, 46]. If the neonatal period is survived, infants continue to have myoclonic and generalized seizures and develop severe dystonic spastic cerebral palsy. Mortality is high due to recurrent lower respiratory tract infections and seizures, with a reported median survival of 3 years [14]. Onset of symptom is later in milder cases, and occasionally children present only with dystonia, spasticity, and a variable degree of developmental delay [7]. The timing of the diagnosis of late onset cases varies, ranging from 16 months to 23 years of age [18, 47].

Variants in the *MOCS1* gene cause two thirds of the reported cases, followed by variants in the *MOCS2* and extremely rare *GPHN* variants. Currently, 40 patients with MoCo deficiency harboring *MOCS2* variants have been reported, most of whom were diagnosed during the neonatal period with intractable initial seizures and feeding disorders [48]. The presenting phenotype in 60% of these subjects is severe and includes facial dysmorphia, intractable neonatal seizures, and multiple cystic cavities in the brain on magnetic resonance imaging. Only three patients presented with a mild clinical phenotype, which manifested as global developmental delay without seizures; however, these cases were lost to follow-up after diagnosis, which was made between birth and 11 months of age [46, 49, 50].

We identified an important association between the homozygous c.244A > T (p.Ile82Phe) *MOCS2* transcript variant 1 (NM_176806.4) and c.57A > T (p.Leu19Phe) *MOCS2* transcript variant 3 (NM_004531.5) and mild xanthinuria type III with later onset in five Roma patients from four families residing in three Eastern European countries with large Roma populations (Czechia, Slovakia, and Macedonia). This mutation is unique as it affects a region in the gene which encodes both subunits of the MPT synthase. In MOCS2A, the small subunit, the C-terminal tail is affected by the p.Ile82Phe exchange. In addition, the other reading frame, which encodes for MOCS2B, the large subunit, the N-terminus will be modified by the p.Leu19Phe variant.

The pathogenicity of the identified MOCS2 variant was confirmed by low concentrations of uric acid in serum, high excretion of xanthine in the urine, and subsequently by low and/or undetectable XO enzyme activity in plasma. The impact of the disorder on the metabolic pathway of sulfur-containing compounds was further demonstrated by significantly low concentrations of total aminothiols (cysteine, homocysteine) and elevated levels of SSC, SSH, and inorganic anions sulfite, thiosulfate, and thiocyanate. However, the levels of sulfite-related metabolites in our patients were not dramatically different from those described in patients with severe MoCD due to *MOCS2* variants and do not explain the mild phenotype or asymptomatic course of the disease [15]. Functional characterization in vitro revealed significantly decreased MPT synthesis activity and thus confirmed the causality of the *MOCS2* c.244A > T variant in MoCD.

Only seven heterozygous alleles of *MOCS2A* c.244A > T have been reported out of a total of 282,880 chromosomes in the ExAc database (accessed Jan 3, 2025). We identified 12 heterozygotes (one in a compound heterozygous patient with renal hypouricemia 1, three in heterozygous carriers with renal hypouricemia 2, and eight in subjects with unknown health status) in a Roma hypouricemia control group of 167 individuals living in the Czech and Slovakia Republics. However, our findings indicate that the c.244A > T variant is quite a frequent variant (allele frequency 3.6%) in the Roma population in Eastern Europe, likely due to endogamy and consanguinity.

The MOCS2 rs776441627 variant is thought to be a pathogenic mutation through in silico prediction. This variant impacts conserved regions in both MPT synthase subunits, MOCS2A and MOCS2B, causing a semiconservative replacement of leucine and isoleucine with a bulky phenylalanine. Based on our biochemical data, p.Ile82Phe shows a stronger impact on complex formation, while p.Leu19Phe is more impactful for MPT synthesis when paired with the respective other WT subunit. However, when both mutant variants are expressed, an additive effect resulting in a 20-fold reduced complex formation was observed, leading to 20% residual MPT synthesis activity. These data underline the structural impact of the phenylalanine substitution in MPT synthase, thus impacting its in vitro activity, with a clearly detectable residual activity that fits the mild phenotype and moderate changes in the biomarker profile.

Population-enriched autosomal recessive diseases are not uncommon in populations where consanguinity is prevalent or that have undergone a founder event, which can arise from geographic or cultural isolation, all of which the Roma have experienced. Moreover, several variants that cause rare diseases are unique to the Roma and have been only recently discovered: e.g., Charcot Marie Tooth disease type 4D and 4G (OMIM 601455 and 605,285), congenital cataract facial dysmorphism neuropathy (OMIM 604166), Gitelman syndrome (OMIM 263800), galactokinase deficiency (OMIM 230200), among others. We found *MOCS2* c.244A > T variant to be present in Czech and Slovak control subjects, all of whom identify as Roma. In a Macedonian sub-cohort, the variant was not found. The Czech and Slovak republics are neighboring countries and between 1918 and 1992 formed one state, Czechoslovakia. A 2010 conference proceeding reported a Spanish child, aged 7, homozygous for the same variant who was asymptomatic for the clinical phenotype but presented with the full biochemical phenotype associated with MoCD [51]. Although Czech and Slovak Roma populations share common history and a similar gene pool, Spanish and also probably Macedonian Roma represent a genetically distant group of Roma [52]. These two groups separated shortly after the arrival of Roma to Europe about 500 years ago, and the differences in presence and frequency of many disease-causing variants were shown by a number of studies [19, 52]. The occurrence of *MOCS2* c.244A > T in the geographically distant Iberian Peninsula is crucial, as it likely indicates a primary founder effect in Roma that would suggest that the variants may be found among all Roma groups deriving from the original founder population. Moreover, we previously published the high frequencies for the common dysfunction allelic variants c.1245_1253del and c.1400C > T of *SLC22A12* (renal hypouricemia type 1, OMIM 220150) among the Roma [53, 54], and those prevalent variants were also identified in Roma Spanish patients with renal hypouricemia [55]. It is therefore necessary to study individual Roma groups to reveal differences in allele frequencies and the prevalence of rare autosomal recessive diseases in Roma subpopulations. Taken together, our data showed the high incidence of variants associated with hypouricemia among the Roma, and this genetic background should be kept in mind during the differential diagnosis.

We believe these findings are important for several reasons. First, MoCo deficiency needs to be considered in patients with biochemical phenotype without severe neurological symptoms. Second, although sulfite and SSC are two well-known biomarkers that can be detected in MoCD patients very shortly after birth, hypouricemia may provide an initial generally available biochemical key marker indicator of MoCo deficiency. We highlight the fact that detailed investigations in patients with unexplained hypouricemia are required. Thirdly, the MOCS2 c.244A > T variant should be considered in Roma hypouricemia patients in the differential diagnosis scheme as it is causal for MoCD type A. Currently, fosdenopterin (cyclic pyranopterin monophosphate) is approved to reduce the risk of mortality in MoCD/MoCo A type patients [56].

In conclusion, we discovered a novel *MOCS2* variant, prevalent in the Roma population, that is associated with xanthinuria and mild clinical phenotypes. Our finding provides new insights for the differential diagnostic algorithm for low serum uric acid and total homocysteine exhibiting clinical signs such as urolithiasis and nephrolithiasis in adolescent or adult patients.

## Supplementary Information

Below is the link to the electronic supplementary material.

Supplementary file1 (PPTX 572 KB)

## Data Availability

No datasets were generated or analysed during the current study.
