# Supplementary material for: A prevalent MOCS2 variant in the Roma population is associated with a novel mild form of molybdenum cofactor deficiency
Source: Eur J Pediatr. 2025 Jul 25;184(8):499. doi: 10.1007/s00431-025-06335-x (PMC12289762; doi:10.1007/s00431-025-06335-x)
Supplement: Supplementary file 1 — Supplementary file1 (PPTX 572 KB) [file 431_2025_6335_MOESM1_ESM.pptx]

## Slide 1
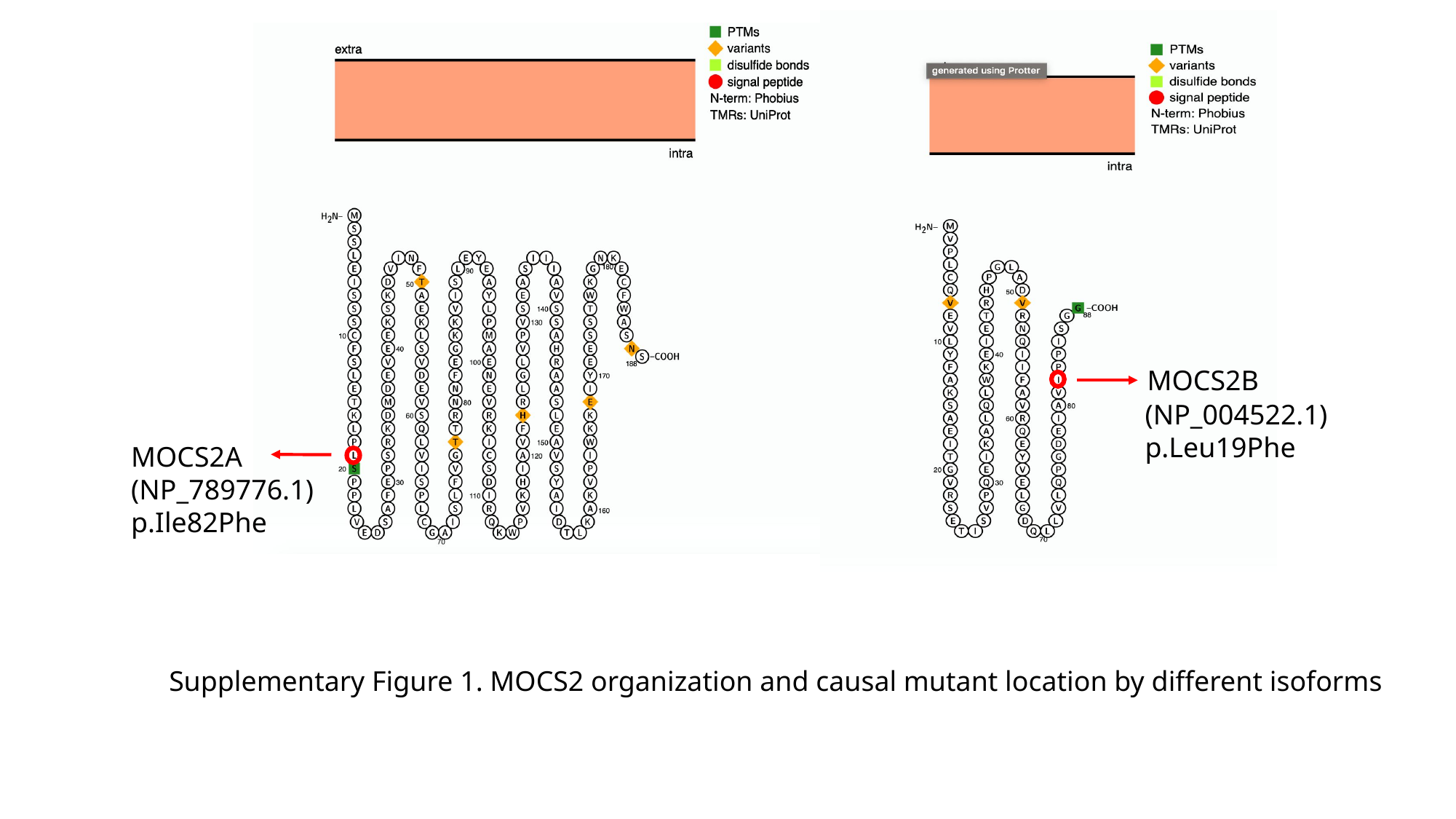

MOCS2B
 (NP_004522.1)
 p.Leu19Phe
 MOCS2A
 (NP_789776.1)
 p.Ile82Phe
Supplementary Figure 1. MOCS2 organization and causal mutant location by different isoforms
